# The continuation of clinical trials in times of war: A need to develop ethics and situationally adaptive clinical research guidelines

**DOI:** 10.3389/fmed.2022.966220

**Published:** 2022-09-16

**Authors:** Chieko Kurihara, Francis P. Crawley, Varvara Baroutsou, Sander Becker, Brigitte Franke-Bray, Courtney A. Granville, Kotone Matsuyama, Shehla Naseem, Johanna Schenk, Sandor Kerpel-Fronius

**Affiliations:** ^1^Kanagaawa Dental University, Kanagawa, Japan; ^2^Good Clinical Practice Alliance - Europe (GCPA) and Strategic Initiative for Developing Capacity in Ethical Review (SIDCER), Leuven, Belgium; ^3^Consultant, Pharmaceutical Medicine, Athens, Greece; ^4^Consultants in Pharmaceutical Medicine, Dover Heights, NSW, Australia; ^5^Independent Consultant, Basel, Switzerland; ^6^Scientific Affairs, Drug Information Association, Washington, DC, United States; ^7^Department of Health Policy and Management, Nippon Medical School, Tokyo, Japan; ^8^Academic and Research College of Family Medicine Pakistan, Karachi, Pakistan; ^9^PPH plus GmbH & Co. KG, Hochheim am Main, Germany; ^10^Department of Pharmacology and Pharmacotherapy, Semmelweis University, Budapest, Hungary

**Keywords:** research ethics, human rights, international humanitarian law, protection of vulnerable study participants, research integrity, adaptive design

## Introduction

Clinical scientists and ethicists have reviewed the unique situation of a large number of clinical trials in Ukraine, Russia, and the region now seriously disrupted by the war. The International Federation of Associations of Pharmaceutical Physicians and Pharmaceutical Medicine (IFAPP) immediately condemned the war with its terrible impact on human health, including that on clinical trials (1). The IFAPP echoed the demand for medical neutrality put forward by the World Medical Association (WMA) (2). Humanitarian and professional organizations as well as ordinary people have generously provided essential support for war victims and refugees. International pharmaceutical manufacturers' associations have expressed their “*mission of providing treatments and vaccines to all those affected by war, wherever they are*” (3). The member companies of The European Federation of Pharmaceutical Industries and Associations (EFPIA) have provided over 22 million doses of essential medicines and more than 62 million Euros of financial support to NGOs (as of August 2022) (4).

## Current status and policy implications

### The continuation of clinical trials in times of crisis

The provision of essential healthcare to those finding themselves in situations of conflict must be a first priority. The Ethics Working Group of the IFAPP have stressed the importance of continuing, where and as possible, the investigational treatments offered to patients already enrolled in clinical trials in countries stricken by various types of disruptive catastrophes (5, 6). There is now a need to review our understanding of clinical research in the context of crisis situations, taking into account a broader appreciation of what must be included in the fundamental right to health (7) as well as the emerging positive interpretation of the rights of study participants to access to potentially vital interventions provided through clinical research and possibly “identified as beneficial in the trial” (8).

We also need to consider that the destructive impact of war and other crisis on healthcare, science, and economy of a society should be fought against and limited as much as possible on all the goods and enterprises of a society. It would be entirely unacceptable that, due to war, those seeking peace and the reconstruction of those impacted would abandon clinical research (including its science and ethics). The pursuit of health and the essential contributions of science to public health, locally and globally, should not be simply given up to the political failures that bring about war and its continuation.

### Study participants beyond the borders

This war presents an unprecedent situation. Hundreds of clinical trials are being conducted in Ukraine where at least seven pharmaceutical companies have been confronted with patient enrollment disruptions and the closure of sites [As of March 2022, (9)]. Our search in ClinicalTrials.gov in the middle of May found 247 phase 3 ongoing (recruiting, not yet recruiting) trials in Ukraine, with 30 located in Kiev (in the middle of August, 216 phase 3 ongoing and 30 in Kiev). There are various therapeutic areas, including placebo-controlled trials. Some pharmaceutical companies are trying to remedy the disruptions in Ukraine by accepting refugee Ukrainian study participants at sites in Poland, Slovakia, Romania, and elsewhere (9). European regulators, including Austria (10) and the European Clinical Trials Cooperation Group (11) have provided brief guiding documents for transferring participants from Ukraine to other EU/EEA countries in case individual benefit of a patient-participant is assumed. Individual companies and trial sites have also taken measures to relocate displaced study participants to other sites within Ukraine as well as in nearby countries. These guidance documents suggest necessary procedures in terms of communications among regulatory authorities, sponsors, clinical trial sites; availability of insurance or interpretation services; transferring already acquired documents, and so on. Extensive international or trans-regional collaboration is needed with destroyed traffic routes and difficulties in electronic data transfer.

A group of clinical research professionals and ethicists, within Ukraine and internationally, came together in April 2022 to examine and support Ukrainian clinical research. This early engagement has led to the development of the Ukrainian Clinical Research Support Initiative (UCRSI). UCRSI has provided a unique platform for responding to urgent questions regarding the support of clinical trial participants, investigators, sites, supply of trial medications, and safety issues as well as addressing Good Clinical Practice and ethics questions as they arise. As a key partner in the UCRSI, the IFAPP has provided contextual support for assuring an appropriate response to the needs of clinical trial participants as well as the maintenance of Ukraine's scientific and health investment in clinical research (12). The disruption in study medications, the abrupt on the ground impacts on clinical staff, the need for ancillary care, diagnostic tools, data management procedures, and above all care for the physical and mental distress of refugees have been a continuing focal point. As the European Medicines Agency suggested, patients' safety must be the first priority and war-related events affecting clinical trials should be assessed according to existing guidelines, with attention to the context-based flexibilities learnings from the COVID-19 pandemic, when considering protocol deviations, missing data, or other conduct or analysis issues in terms of applications for marketing approval (13).

### Revising “access to clinical trials” and “protecting the most vulnerable” in terms of “situational adaptive design”

This situation requires us to face the critical point of reconstructing our theories and frameworks supporting the protection of vulnerable study participants, those in war or conflict settings. Traditionally, we have excluded vulnerable populations from clinical research, such as captive soldiers, refugees, and those affected by natural disasters. The recent landscape of research ethics now moves toward promoting the inclusion of vulnerable populations (14). This premise was demonstrated previously in the HIV/AIDS pandemic in the 1980s with controversy on the right of access to trials or safety risks (15), and again most recently in the crisis situations arising in the context of the Ebola epidemic and then the COVID-19 pandemic. These contexts for clinical research repeat themselves similarly in natural disasters and war conflicts. The CIOMS ethical guidelines for health-related research stated that studies in disaster situations should be conducted as “*an integral part of disaster response*”. At times researchers are confronted with complex obligations: health professionals are expected to, even obliged to, provide humanitarian support; at the same time, they will want to ensure that this support being provided is by the most appropriate, most efficient, and safest methods possible—and this may well require observational or interventional research. In such disruptive situations, patient autonomy is fragile, their situation creates new vulnerabilities and new dependencies (14). Nonetheless healthcare and other humanitarian actions are an essential need, and these actions require to be studied if they are to be the best possible, if we are to avoid past mistakes, and if the global healthcare and humanitarian communities are to improve their emergency preparedness and response methodologies.

In the context of our ongoing discussion on clinical trials in Ukraine during this war, we have also been able to shed new light on the “therapeutic misconception” (16), which can be simply explained: patients may seek for benefits of experimental therapy without sufficient understanding of the risks of experimentation (17). The disruptiveness and urgency of the war requires each investigator to anew evaluate each clinical trial participant's health situation, the required care, and the extent to which ongoing participation in the clinical trial is the only or best way to address their health needs. It is a premise that in conflict setting limited resource must be re-allocated prioritizing essential health care. In this context, continuation or discontinuation of each trial project and each patient's participation needs extremely careful, difficult decision considering healthcare of each patient as well as local, catastrophic situations. Governmental decision may be needed for protection from attack by the hostile forces, meanwhile, political interest of authority may contradict humanitarian motive of research team (18). The war has, inadvertently, required us to re-evaluate the real value of clinical trials, individually and as an enterprise in healthcare provision, alongside vulnerabilities and patient demands. The war has brought into perspective the need to prioritize health and science above all private or commercial interests. Ukraine is an unfortunate yet stark example as to just why our patients, our societies require clinical research. By paying clear attention to the therapeutic misconception, not simply as an abstract concept but (more importantly) as a real threat that is also context dependent, we are also confronted with the real value of clinical research for individuals, for society. Ukrainian healthcare professionals and investigators and other research in war zone suggest us that clinical research is not a commercial luxury; clinical research is a health necessity.

In some situations, investigational medicines could, and should, be prioritized as a part of humanitarian expanded access programs, outside the research protocol. Here we need adaptable research designs that are situationally dependent and context driven rather only developed as part of the process of speeding up medicines development, also in order not to waste the valuable clinical data generated owing to the altruism of the participants. We need to rethink and redesign our “adaptive design” models for clinical trials such that they are also fit for purpose for war, crisis, and disaster situations, sometimes including go/no-go decision algorithm in extremely difficult situations. In cases where study protocols are fundamentally disrupted, adaptive design should mean adapting to the needs of study participants—not abandoning the participants, not abandoning the health intervention, not abandoning the science. It may well mean suspending the commercial or marketing interests, but this is something we should be prepared to do, also for the benefit of future commercial and marketing interests. This concept of “situational adaptive design” may be included in the original study design in advance of initiation of study when this study is essential as a part of disaster response, or otherwise possible to be applied when unexpected crisis happened in the middle of conduct of study and continuation of this study is concluded to be essential at this specific situation.

While safety concerns do arise for the use of experimental medicines outside the trial protocol (19), risk minimization strategies should be developed according to the “monitored emergency use of unregistered and experimental intervention” (MEURI) framework, proposed by the World Health Organization for disease outbreak situations (20). The rigorous safety monitoring of each patient is prerequisite, and the results of observational data should be consolidated with other trial data, considering the differences among subgroups due to the changing background situations. There could be the case that we should supply alternative interventions instead of investigational products. Access to care must be assured among the study participants and other people in resource-destroyed areas. All of this, however, requires a new adaptive, situationally adaptive, approach to clinical trials ensuring that, even with severe changes in the possibilities to exercise a protocol, the scientific approach to healthcare and the evaluation of interventions is continued. Considering all these aspects, we need to establish a revised ethics foundation for the protection of research participants in times of war, assuring their health needs while simultaneously maintaining scientific integrity.

### Clinical research at the intersection of humanitarian and human rights laws

Recently, the crossroad between humanitarian law and human rights law appears to be expanding. International humanitarian law expresses moral exigencies in times of war; human rights law expresses moral requirements largely developed as a way to protect individuals in times of peace. Both legal frameworks have evolved independently while expressing common values for the protection of life, health, and human dignity (21). Research ethics documents have been developed in settings where international human rights law is also brought into play. CIOMS 2016 guidelines is the first international document to provide comprehensive guidance on the protection of research participants in environments of war or conflict, which is one of the disaster situations.

The Geneva Convention, the basis of humanitarian laws, prohibits attacks on injured soldiers, prisoners of war, civilians, hospitals, and health workers, while also permitting medical or scientific experiments on prisoners of war only when this is in the interest of the study subjects (22). Its commentary addresses the war crimes involving human experimentation by Nazi and Japanese imperial military (23). On the other hand, international human rights law has been developed from the Universal Declaration of Human Rights of the United Nations in 1948 (24) where there is a direct concern never to allow again atrocities such as those that happened in the Second World War. This Universal Declaration proclaims fundamental rights belonging to the entire human family, focusing on freedoms and justice as guarantees of stability and peace in society. This has then been reiterated in the International Covenants of Human Rights in 1996, including only one clause of research ethics, the prohibition of scientific experiments without consent (25). These two international treaties established the role of international organizations to ensure the protection of human rights in wartime and peacetime. However, these are not sufficient international legal framework to provide protection for both of research participants and medical professionals engaged in research to generate scientific knowledge providing essential care for research participants.

The World Medical Association (WMA)'s Declaration of Helsinki (DoH) (8) states to prioritize patient's right and benefit to the goal of research, based on the immediate post war statements of physicians' obligations (26, 27), reflecting physicians' “War Crimes and Medicine” (28). With the recognition of the WMA that “*Medical ethics in times of armed conflict is identical to medical ethics in times of peace*” (29), the DoH has been developed in the settings of human rights law. Meanwhile, the WMA has also developed medical ethics framework in the humanitarian law settings to protest against wars and use of inhuman weapons (30–35), without deliberation on ethics of medical experimentation in war setting, and now reached to resolution to support Ukrainian medical personnel and citizens (36). Research ethics community has not yet sufficiently discussed maturely enough to reach an international consensus on sensitive issues, e.g., the legitimacy of experimental treatments for prisoners of war, research on the effects of inhumane weapons. The social value generated from such research must ensure the principle of no tolerance for inhumanity during war time. This background suggests to us the need to rethink and expand our research ethics framework, recognizing the important public health contribution of the ongoing pursuit of scientific knowledge and research into the best medical interventions across all human settings, including those of war and other humanitarian or natural disasters.

## Recommendations and conclusions

The war in Ukraine, due to the high number of clinical trials that are ongoing and the importance of clinical research to so many patients and to public health in the region, requires us to re-evaluate our fundamental ethics frameworks as well as the overall role played by clinical research in society.

We need a revised ethics framework as well as a revised situationally adaptive approach to clinical research that is appropriate to the needs of patients and society in times of war and other fundamental disruptions. This framework and the overall approach to clinical research is required if we are to ensure our adherence to patient safety, public health, and the important contexts of humanitarian and human rights legal frameworks.

Hence, refining the research ethics framework primarily developed for peaceful settings and developing situationally adaptive frameworks to war settings and other disasters should now be an essential mission for scientists, clinical research professionals, patient organizations, and bioethicists.

## Author contributions

CK, FC, and SK-F: substantial contribution to the conception or design of the work, or the acquisition, analysis or interpretation of data for the work, and revising it critically for important intellectual content. All authors contributed both to the development of the ideas as well as to the writing of the manuscript and approved the submitted version.

## Conflict of interest

Author SN was employed by Ferozsons Laboratories Ltd. Author JS is owner and executive consultant of PPH plus GmbH & Co. KG.

The remaining authors declare that the research was conducted in the absence of any commercial or financial relationships that could be construed as a potential conflict of interest.

## Publisher's note

All claims expressed in this article are solely those of the authors and do not necessarily represent those of their affiliated organizations, or those of the publisher, the editors and the reviewers. Any product that may be evaluated in this article, or claim that may be made by its manufacturer, is not guaranteed or endorsed by the publisher.
